# Activity-dependent regulation of NMDA receptors in substantia nigra dopaminergic neurones

**DOI:** 10.1113/jphysiol.2013.267310

**Published:** 2014-01-31

**Authors:** Angela R Wild, Susan Jones, Alasdair J Gibb

**Affiliations:** 1 Department of Neuroscience, Physiology & Pharmacology, University College LondonGower Street, London WC1E 6BT, UK; 2 Department of Physiology, Development & Neuroscience, University of CambridgeDowning Street, Cambridge CB2 9JR, UK

## Abstract

*N*-Methyl-d-aspartate receptors (NMDARs) are Ca^2+^-permeable glutamate receptors that play a critical role in synaptic plasticity and promoting cell survival. However, overactive NMDARs can trigger cell death signalling pathways and have been implicated in substantia nigra pars compacta (SNc) pathology in Parkinson's disease. Calcium ion influx through NMDARs recruits Ca^2+^-dependent proteins that can regulate NMDAR activity. The surface density of NMDARs can also be regulated dynamically in response to receptor activity via Ca^2+^-independent mechanisms. We have investigated the activity-dependent regulation of NMDARs in SNc dopaminergic neurones. Repeated whole-cell agonist applications resulted in a decline in the amplitude of NMDAR currents (current run-down) that was use dependent and not readily reversible. Run-down was reduced by increasing intracellular Ca^2+^ buffering or by reducing Ca^2+^ influx but did not appear to be mediated by the same regulatory proteins that cause Ca^2+^-dependent run-down in hippocampal neurones. The NMDAR current run-down may be mediated in part by a Ca^2+^-independent mechanism, because intracellular dialysis with a dynamin-inhibitory peptide reduced run-down, suggesting a role for clathrin-mediated endocytosis in the regulation of the surface density of receptors. Synaptic NMDARs were also subject to current run-down during repeated low-frequency synaptic stimulation in a Ca^2+^-dependent but dynamin-independent manner. Thus, we report, for the first time, regulation of NMDARs in SNc dopaminergic neurones by changes in intracellular Ca^2+^ at both synaptic and extrasynaptic sites and provide evidence for activity-dependent changes in receptor trafficking. These mechanisms may contribute to intracellular Ca^2+^ homeostasis in dopaminergic neurones by limiting Ca^2+^ influx through the NMDAR.

## Introduction

Dopaminergic neurones of the substantia nigra pars compacta (SNc) are critically involved in the selection and learning of voluntary motor behaviour (Groenewegen, 2003; Morikawa & Paladini, 2011). The degeneration and loss of these neurones gives rise to severe motor impairment and disability, which are defining features of Parkinson's disease (Braak *et al*. 2003; Obeso *et al*. 2010; Surmeier *et al*. 2010). While the origins of the neuronal pathology underlying Parkinson's disease are not yet clear, dysregulated Ca^2+^ and mitochondrial dysfunction may have a prominent role (Surmeier *et al*. 2010, 2011). Elevated intracellular [Ca^2+^] can activate signalling pathways coupled to cell death (Hardingham & Bading, 2010; Surmeier *et al*. 2010), has a detrimental effect on mitochondrial function (Duchen *et al*. 2008) and enhances the toxic effects of a high cytosolic dopamine concentration (Mosharov *et al*. 2009). Through one or more of these mechanisms, perturbations in Ca^2+^ homoeostasis may contribute to nigrostriatal degeneration.

One source of Ca^2+^ influx is through *N*-methyl-d-aspartate receptors (NMDARs). Overactivity of NMDARs and resultant elevated intracellular [Ca^2+^] can lead to neuronal excitotoxicity (Rothman & Olney, 1986; Choi, 1987; Hardingham & Bading, 2010) and has been implicated in the pathogenesis of Parkinson's disease (Sonsalla *et al*. 1998; Blandini *et al*. 2000), and NMDA reduces dopamine cell density in SNc (Wild *et al*. 2013). Nigrostriatal degeneration also results in increased glutamatergic output from the subthalamic nucleus to the SNc (Blandini *et al*. 2000; Hamani *et al*. 2004). Convergence of enhanced glutamatergic input with pathologically potentiated NMDAR activity might elevate intracellular [Ca^2+^] beyond homeostatic control (Blandini *et al*. 2000; Surmeier *et al*. 2011).

The NMDARs are themselves regulated by an array of Ca^2+^-dependent and Ca^2+^-independent proteins (Dingledine *et al*. 1999; Nong *et al*. 2003; Salter *et al*. 2009; Traynelis *et al*. 2010) that control the sensitivity of neurones to glutamate, shape synaptic NMDAR responses, impact on synaptic plasticity mechanisms and potentially regulate excitotoxicity (Tong *et al*. 1995; Sprengel *et al*. 1998; Hashimoto *et al*. 2002; Sessoms-Sikes *et al*. 2005). The extent to which these mechanisms operate in SNc dopaminergic neurones is not known, despite the potential clinical implications of dysregulated Ca^2+^ influx.

The purpose of this study was to investigate mechanisms of activity-dependent regulation of NMDARs in dopaminergic neurones. NMDARs activated by whole-cell applications of NMDA were negatively regulated by Ca^2+^ influx through NMDARs in an activity-dependent manner. However, this Ca^2+^-dependent run-down was not attenuated by intracellular dialysis with a calmodulin-inhibitory peptide or inhibitors of calcineurin or actin depolymerization, three Ca^2+^-dependent mechanisms important in cortical and hippocampal neurones. The NMDARs found in SNc dopaminergic neurones were less sensitive to Ca^2+^-dependent run-down than NMDARs found on neighbouring non-dopaminergic neurones. Run-down of whole-cell NMDAR responses was attenuated by a dynamin-inhibitory peptide, suggesting that run-down is partly dependent on receptor trafficking. During repetitive synaptic stimulation, the amplitude of synaptic NMDAR EPSCs in dopaminergic neurones decreased in a Ca^2+^-dependent but dynamin-independent manner. Thus, both synaptic and extrasynaptic NMDARs in SNc dopaminergic neurones show robust and, in some aspects, distinct activity-dependent self-regulation. These mechanisms could be important in Ca^2+^ homeostasis in dopaminergic neurones.

## Methods

### Brain slice preparation

Sprague–Dawley or Wistar rats (aged 5–8 days postnatal) were deeply anaesthetized with isofluorane or halothane and/or decapitated in accordance with the UK Animals (Scientific Procedures) Act 1986 and Local Ethical Committee approval. One hundred and fifteen rats were used in this study. The brain was removed into ice-cold oxygenated slicing solution containing (mm): sucrose, 100; NaCl, 62.5; KCl, 2.5; CaCl_2_, 1; MgCl_2_, 4; NaHCO_3_, 25; NaH_2_PO_4_, 1.25; 0.1 mM kynurenic acid and glucose, 25; of pH 7.4 when saturated with 95% O_2_ and 5% CO_2_. Coronal or horizontal brain slices (300 μm thick) were prepared using a vibrating microslicer (Dosaka, Japan or Leica, Germany). Slices containing the midbrain substantia nigra region were kept in oxygenated solution containing (mm): NaCl, 125; KCl, 2.5; CaCl_2_, 1; MgCl_2_, 4; NaHCO_3_, 25; NaH_2_PO_4_, 1.25; and glucose, 25 for 1–6 h before use.

### Whole-cell recordings from substantia nigra neurones

Slices were placed in a recording chamber on the stage of an upright microscope with Nomarski differential interference contrast optics. During whole-cell current recordings, coronal slices were continuously bathed in the same solution used for storing the slices, without added MgCl_2_ and with bicuculline (10 μm), strychnine (10 μm) and tetrodotoxin (100 nm; from Ascent Scientific) at room temperature (22–24°C). Synaptic currents were recorded from horizontal slices in 6,7-dinitro-quinoxaline-2,3-dione (DNQX; 10 μm), picrotoxin (50 μm) and with 0.1 or 1.3 mm MgCl_2_ at 30 ± 2°C. Patch pipettes were made from borosilicate glass to a final resistance of 2–6 MΩ when filled with solution containing (mm): CsCl, 140; Hepes, 10; NaCl, 10; CaCl_2_, 0.5; MgCl_2_, 0.5–1.0; and CsOH, 29.53; pH 7.2, with Na_2_ATP, 2; NaGTP, 0.3 (all from Sigma); and Ca^2+^ chelators (EGTA or BAPTA) or other additions as specified in the text. Free Ca^2+^ was calculated using WinMAXC32.exe (http://maxchelator.stanford.edu/downloads.htm).

Recordings were made from visually identified neurones in the SNc (unless otherwise noted), identified based on the presence of a time-dependent, hyperpolarization-activated inward current (*I*_h_) of more than 50 pA in response to a voltage step from −60 to −110 mV (Washio *et al*. 1999; Neuhoff *et al*. 2002; Margolis *et al*. 2006). Over 90% of neurones in the rat SNc are dopaminergic (Fallon & Loughlin, 1995). Neurones in the nearby substantia nigra pars reticulata (SNr) were recorded in some experiments; these are primarily GABAergic projection neurons.

Cells were voltage clamped to −60 or +40 mV as specified in the text, and whole-cell currents or synaptic currents were recorded using an Axopatch 200B patch-clamp amplifier (Axon Instruments, USA), filtered at 2 kHz and digitized at 10 kHz using a Micro 1401 interface (Cambridge Electronic Design, Cambridge, UK). Cell membrane capacitance and series resistance were estimated using the series resistance compensation circuit of the Axopatch 200B (series resistance was compensated by 40–80%) and checked throughout the experiment for stability. Whole-cell NMDA currents were evoked by adding NMDA (200 μm; Sigma) and glycine (10 μm; Sigma) by switching to agonist solution for 15 s using a solenoid valve system (c-Flow; Cell MicroControls, Norfolk, VA, USA) to regulate solution flow to a theta-glass applicator placed ∼1 mm above the surface of the slice. These responses had 10–90% rise times of 3–5 s (Suárez *et al*. 2010). Synaptic currents were evoked using a bipolar stainless-steel electrode (Frederick Haer and Co., USA) and stimuli (100 μs duration; amplitude 60–250 μA) applied at 10 s intervals.

### Electrophysiological measurements

Whole-cell NMDA currents were acquired and analysed using WinEDR version 2.5.9 software (Dempster, 2001; version 2.2.3 available at http://spider.science.strath.ac.uk/PhysPharm/showPage.php?pageName=software_ses). Synaptic currents were acquired and analysed using Spike2 (version 4) software (Cambridge Electronic Design, Cambridge, UK). To quantify run-down in responses to consecutive agonist applications in the different conditions tested, the decrease in peak *I*_NMDA_ between the first and *n*th NMDA application was measured. To quantify run-down of synaptic NMDAR responses over comparable time scales to agonist-evoked responses, nine responses to synaptic stimulation (given every 10 s) were averaged (e.g. ‘time 0 s’ is the average of nine responses from 0 to 90 s) and the decrease in mean peak NMDA-EPSC amplitude between the first and fifth time point at 400 s was measured. Data are expressed as means ± SEM; the ‘*n*’ values refer to the number of slices. For all data sets, normality was tested using the Kolmogorov–Smirnoff test (GraphPad Prism version 4). For comparisons of two groups of data, Student's *t* test was used (with Welch's correction if variance between the groups was different) and for more than two groups of data ANOVA followed by Bonferroni multiple comparisons test was used.

## Results

### NMDARs in SNc dopaminergic neurones show activity-dependent run-down

The NMDAR-mediated whole-cell responses show run-down with repeated applications of NMDA to SNc dopaminergic neurones (Suárez *et al*. 2010). In order to investigate regulatory mechanisms that might mediate this effect, NMDA (200 μm, with 10 μm glycine, for 15 s) was applied repeatedly every 100 s. As illustrated in Fig. 1*A*, in dopaminergic neurones voltage clamped to −60 mV and with 10 mm BAPTA in the pipette solution, following an initial application of NMDA, there was a subsequent decline in peak current with each successive application, which we define here as current ‘run-down’. The first response to NMDA (*I_t_*_0_) had an average amplitude of −4334 ± 296 pA, while the fifth response (*I_t_*_400_) was significantly less and averaged −2558 ± 235 pA (*P* < 0.0001, Student's paired *t* test, *n* = 16; Fig. 1*B*). The *I_t_*_400_/*I_t_*_0_ ratio, used to quantify the extent of run-down, was 0.59 ± 0.03 (Fig. 1*E*). To determine whether the observed run-down was activity dependent, in a separate group of neurones NMDA was applied every 400 s (Fig. 1*C*). In these cells, *I_t_*_400_ was not significantly different from *I_t_*_0_ (Fig. 1*D* and *E*; *P* = 0.22, Student's paired *t* test, *n* = 8), with a ratio of 1.17 ± 0.09. Significantly more run-down was seen at *I_t_*_400_ when NMDA was applied every 100 s than when applied every 400 s (Fig. 1*E*; *P* < 0.006, Student's unpaired *t* test with Welch's correction), suggesting that repeated receptor activation or current through the receptor channel leads to run-down.

**Figure 1:**
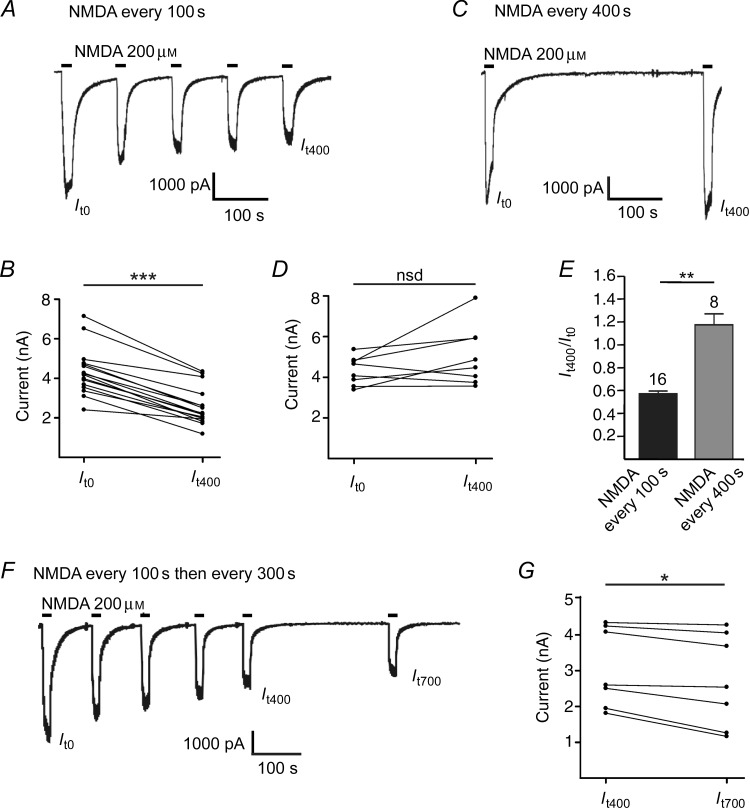
*A*, representative current trace showing responses to repeated brief applications of 200 μm NMDA (glycine, 10 μm) applied every 100 s. *B*, graph comparing current amplitudes after one NMDA application (*I_t_*_0_) with those after five applications (*I_t_*_400_) from 16 experiments as in *A* (***P < 0.001, Student's paired *t* test). *C*, example current trace as in *A* but with NMDA applications every 400 s. *D*, comparison of current amplitudes after one NMDA application (*I_t_*_0_) with those after two applications (*I_t_*_400_) from eight experiments as in *C*. *E*, ratio of *I_t_*_400_/*I_t_*_0_ for NMDA applications every 100 and every 400 s (**P = 0.006, Student's unpaired *t* test with Welch's correction). *F*, representative current trace showing responses to 200 μm NMDA (glycine, 10 μm) applied every 100 s, then after 300 s *G*, paired current amplitudes after five NMDA applications (*I_t_*_400_) and those after six applications (*I_t_*_700_) from seven experiments as in *F* (*P < 0.05, Student's paired *t* test). All experiments were performed at a holding potential of –60 mV with 10 mm BAPTA in the pipette solution.

Modifications to NMDARs that may mediate current run-down in dopaminergic neurones, such as changes in receptor phosphorylation or association of regulatory proteins, can be transient; therefore, we tested the ability of NMDARs in dopaminergic neurones to recover from run-down. Following five repeated applications of NMDA every 100 s, a pause of 300 s was allowed (Fig. 1*F* and *G*) to determine whether recovery from run-down would occur. There was a small but significant decrease in current amplitude at 700 s (−2721 ± 491 pA) compared with 400 s (−3076 ± 419; *n* = 7; *P* = 0.0114, Student's paired *t* test), indicating that the run-down persists and is not readily reversible in these whole-cell recording conditions.

### Run-down of whole-cell NMDAR responses is in part Ca^2+^ dependent

Calcium-dependent run-down is a well-established form of NMDAR regulation (Legendre *et al*. 1993; Rosenmund & Westbrook, 1993a,*b*1993b; Tong & Jahr, 1994; Ehlers *et al*. 1996; Medina *et al*. 1996; Rycroft & Gibb, 2002); therefore, a series of experiments was designed to assess the dependence of NMDAR current run-down on changes in intracellular Ca^2+^ concentration. First, two different Ca^2+^ buffers were compared to investigate the effect that sequestering intracellular Ca^2+^ would have on the amplitudes of NMDAR responses. A high concentration of fast Ca^2+^ buffer (10 mm BAPTA, estimated free Ca^2+^ ∼20 nm) was compared with a low concentration of slow Ca^2+^ buffer (0.6 mm EGTA, estimated free Ca^2+^ ∼735 nm). Changing the Ca^2+^ chelator in the intracellular solution from 10 mm BAPTA (e.g. Fig. 1*A*) to 0.6 mm EGTA (e.g. Fig. 2*A*) had no effect on the initial current amplitude (*I_t_*_0_ = −4644 ± 462 pA, *n* = 17 in 0.6 mm EGTA; Supporting information Table S1) but resulted in more run-down (*I_t_*_400_/*I_t_*_0_) in dopaminergic neurones voltage clamped to −60 mV (EGTA, *I_t_*_400_/*I_t_*_0_ = 0.37 ± 0.02, *n* = 15; BAPTA, 0.59 ± 0.03, *n* = 16; *P* < 0.001, ANOVA with Bonferroni multiple comparisons test; Fig. 2*B* and *C*).

**Figure 2:**
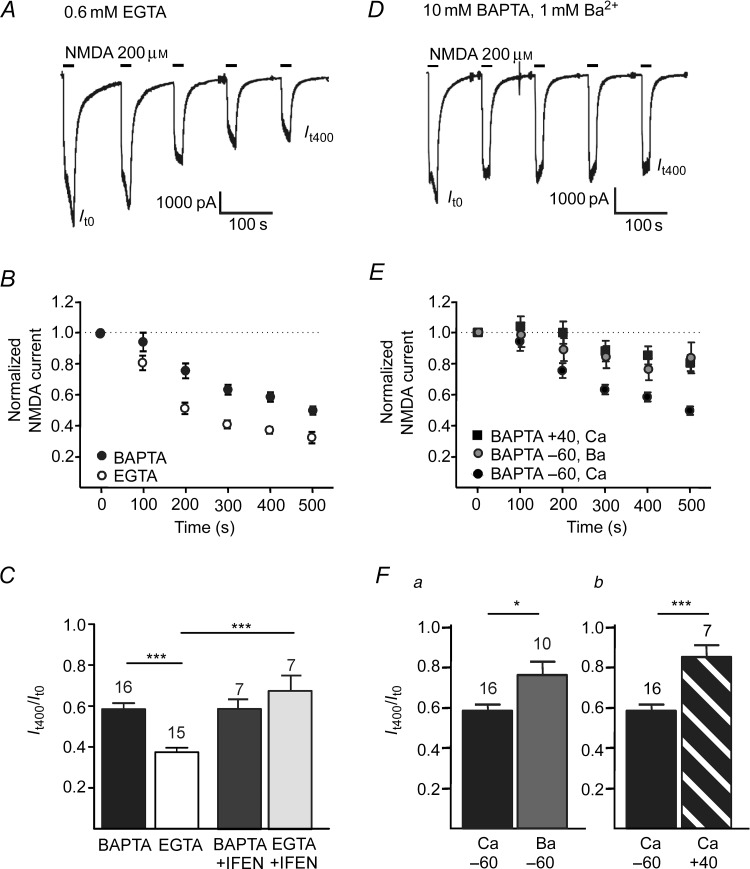
*A*, responses to repeated brief applications of 200 μm NMDA (glycine, 10 μm) applied every 100 s with 0.6 mm EGTA in the pipette solution. *B*, time course of NMDA current run-down with either 0.6 mm EGTA or 10 mm BAPTA in the pipette solution. Current amplitudes are normalized to the amplitude of the first response. *C*, ratio of *I_t_*_400_/*I_t_*_0_ with 0.6 mm EGTA or 10 mm BAPTA in the pipette solution for the full component and for the ifenprodil-insensitive component (***P < 0.001, ANOVA with Bonferroni multiple comparisons test). *D*, as in *A*, but with 10 mm BAPTA in the pipette solution and extracellular Ca^2+^ replaced with equimolar Ba^2+^. *E*, time course of NMDA current run-down with 10 mm BAPTA in the pipette solution at –60 mV, extracellular divalent cation substitution with Ba^2+^ at –60 mV or with Ca^2+^ at +40 mV. Current amplitudes are normalized to the average amplitude of the first response. *F*, ratio of *I_t_*_400_/*I_t_*_0_ with 10 mm BAPTA in the pipette solution, at –60 mV with extracellular divalent cation either Ca^2+^ or Ba^2+^ (*a*) and with membrane potential clamped to –60 or to +40 mV (*b*; *P = 0.025; **P < 0.003, ANOVA with Bonferroni multiple comparisons test).

To determine whether this Ca^2+^ dependence was NMDAR subunit dependent, the experiments were repeated following incubation with the GluN2B-preferring antagonist, ifenprodil (10 μm; minimal incubation time, 30 min). Interestingly, run-down was not significantly different between BAPTA (0.59 ± 0.05, *n* = 7) and EGTA (0.67 ± 0.08, *n* = 7; *P* > 0.05, ANOVA with Bonferroni multiple comparisons test; Fig. 2*C*), suggesting that run-down of the non-GluN2B component is not Ca^2+^ dependent.

Potential sources of Ca^2+^ that could induce current run-down are that which has fluxed through the channel pore or that which has been released from intracellular stores (e.g. by Ca^2+^-induced Ca^2+^ release). Extracellular Ca^2+^ was replaced with equimolar Ba^2+^ (e.g. Fig. 2*D*). With 10 mm BAPTA in the pipette, following substitution of extracellular Ca^2+^ with Ba^2+^ there was still significant run-down (*I_t_*_400_/*I_t_*_0 _= 0.70 ± 0.04, *n* = 10; *P* = 0.0054, Student's paired *t* test) but significantly less than that seen with 1 mm extracellular Ca^2+^ (*P* = 0.025, Student's unpaired *t* test; Fig. 2*E* and *F*).

The driving force for Ca^2+^ moving into the cell was reduced by voltage clamping the neurone at +40 mV. At +40 mV (with 10 mm BAPTA in the pipette solution), there was no significant change in peak current between the first and fifth response (average amplitude of first response, 3911 ± 431 pA, Table S1; *P* = 0.0581, Student's paired *t* test) and the *I_t_*_400_/*I_t_*_0_ ratio (0.85 ± 0.06, *n* = 7) was significantly different from that seen at −60 mV (*P* = 0.0026, Student's unpaired *t* test; Fig. 2*E* and *F*). Thus, the amount of current through active NMDARs in dopaminergic neurones is regulated by intracellular Ca^2+^ and is sensitive to the amount of Ca^2+^ influx through the NMDAR channel.

### Regulation of synaptic NMDARs by Ca^2+^

Changes in intracellular Ca^2+^ can also affect the amplitude of NMDA EPSCs over time, because synaptic NMDARs are also regulated in a Ca^2+^-dependent manner (Tong *et al*. 1995; Medina *et al*. 1999; Umemiya *et al*. 2001). In dopaminergic neurones voltage clamped to +40 mV, the initial NMDA-EPSC responses to evoked glutamate release (*I_t_*_0_) had an average amplitude of 50.2 ± 7.8 pA (10 mm BAPTA) or 59.6 ± 10.3 pA (0.6 mm EGTA). The NMDA EPSCs showed a small but significant degree of run-down at +40 mV (e.g. 10 mm BAPTA *I_t_*_400_/*I_t_*_0_, 0.86 ± 0.06, *n* = 10; *P* = 0.0344, Student's paired *t* test; Fig. 3*A*). Run-down was more marked at −50 mV (10 mm BAPTA: initial NMDA-EPSC amplitude, −66.6 ± 7.5 pA; *I_t_*_400_/*I_t_*_0_, 0.58 ± 0.05, *n* = 10; *P* < 0.0001, Student's paired *t* test; Fig. 3*B*). Run-down of NMDA EPSCs was not significantly different from run-down of whole-cell responses to NMDA at +40 mV (*P* = 0.948, Student's unpaired *t* test) or −60 mV (*P* = 0.974, Student's unpaired *t* test) with 10 mm BAPTA in the pipette. However, synaptic NMDARs showed no sensitivity to intracellular Ca^2+^ buffering; at −50 mV, with 0.6 mm EGTA, initial NMDA-EPSC amplitude was −54.2 ± 7.1 pA and *I_t_*_400_/*I_t_*_0_ was 0.67 ± 0.08 (*n* = 8; *P* = 0.0086, Student's paired *t* test), not significantly different from *I_t_*_400_/*I_t_*_0_ for 10 mm BAPTA at −50 mV (*P *> 0.05, ANOVA with Bonferroni multiple comparisons test; Fig. 3*C* and *D*).

**Figure 3:**
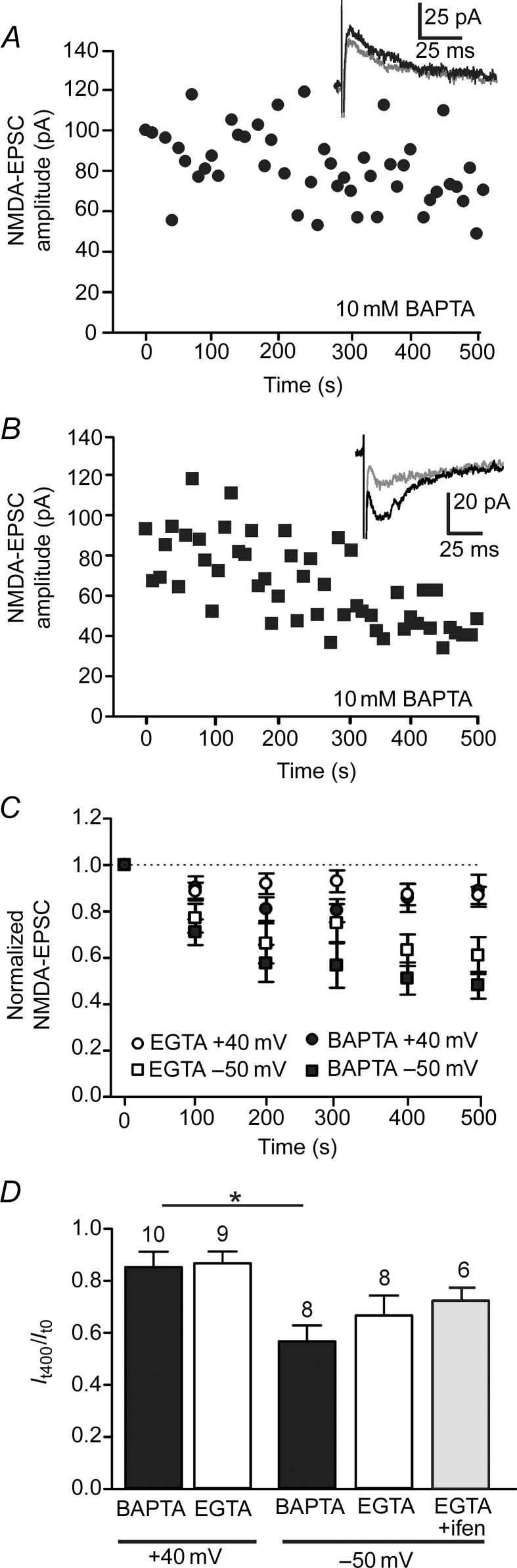
*A*, representative NMDAR EPSCs recorded from a single dopaminergic neurone evoked by synaptic stimulation at 0.1 Hz in the presence of 6,7-dinitro-quinoxaline-2,3-dione (DNQX, 10 μm), glycine (10 μm), picrotoxin (50 μm) and Mg^2+^ (0.1 mm) at +40 mV with 10 mm BAPTA in the pipette solution. Inset traces are the average of 10 EPSCs recorded between 0 and 90 s (black) or between 400 and 490 s (grey). *B*, NMDAR EPSCs evoked by synaptic stimulation as in *A* but at −50 mV (with 10 mm BAPTA). *C*, graph showing changes in NMDAR-EPSC amplitude over time at +40 mV (circles) and −50 mV (squares), with either 0.6 mm EGTA (white symbols) or 10 mm BAPTA (black symbols) in the pipette. Each time point represents the average of 10 EPSCs and is normalized to the average amplitude of the first 10 responses. *D*, ratio of *I_t_*_400_/*I_t_*_0_ with 10 mm BAPTA or 0.6 mm EGTA in the pipette solution, at either +40 or −50 mV, with the latter including ifenprodil-insensitive NMDA EPSCs (*P < 0.05, ANOVA with Bonferroni multiple comparisons test). The *I_t_*_0_ is the average of 10 EPSCs between 0 and 90 s, while the *I_t_*_400_ is the average of 10 responses between 400 and 490 s.

To determine whether NMDA-EPSC run-down at −50 mV was subunit dependent, the experiments were repeated following incubation with the GluN2B-preferring antagonist, ifenprodil (10 μm; minimal incubation time, 30 min). Run-down was not significantly different following incubation in the presence of ifenprodil (0.59 ± 0.05, *n* = 7; *P *> 0.05, ANOVA with Bonferroni multiple comparisons test; Fig. 3*D*).

### Differences in NMDAR regulation between dopaminergic and GABAergic neurones

Dopaminergic neurones of the substantia nigra are more vulnerable to cell death than neighbouring GABAergic neurones in Parkinson's disease (Braak *et al*. 2003). Dysregulated Ca^2+^ homeostasis, including differences in regulation of NMDAR-mediated Ca^2+^ influx, may contribute to this selective vulnerability. Therefore, we next compared the degree of NMDAR run-down in dopaminergic neurones (e.g. Figs 1*A* and 4*B*) with that in GABAergic neurones (e.g. Fig. 4*A* and *B*) with the two levels of intracellular Ca^2+^ buffering. There was no significant difference in the initial amplitudes in GABAergic neurones compared with dopaminergic neurones with either BAPTA or EGTA (*P* = 0.66 and *P* = 0.29, respectively). However, there was significantly more whole-cell NMDAR current run-down in GABAergic neurones (Fig. 4*C*) with BAPTA (0.45 ± 0.05, *n* = 9, *P* < 0.05), but not with EGTA in the intracellular solution, compared with dopaminergic neurones (ANOVA with Bonferroni multiple comparisons test). Run-down of synaptic NMDARs in response to glutamate release at −50 mV was not significantly different between the two neuronal populations with either chelator (ANOVA with Bonferroni multiple comparisons test). Run-down of whole-cell and synaptic NMDARs in dopaminergic *versus* GABAergic neurones is summarized in Fig. 4*C*.

**Figure 4:**
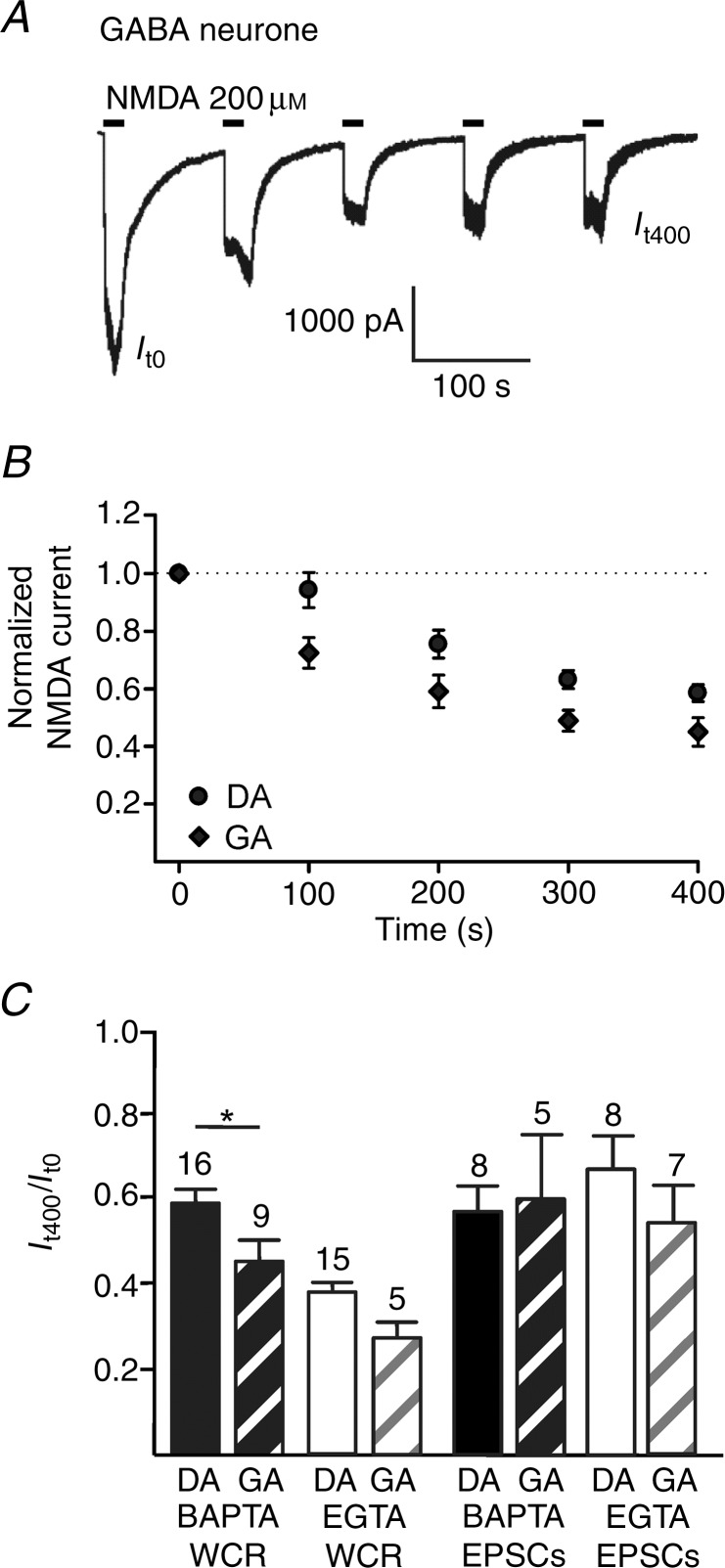
*A*, responses to repeated brief applications of 200 μm NMDA (glycine, 10 μm) applied every 100 s with 10 mm BAPTA in the pipette solution, from a GABAergic neurone. *B*, time course of NMDAR current run-down with 10 mm BAPTA in the pipette solution for dopaminergic (DA) and GABAergic neurones (GA). Current amplitudes were normalized to the amplitude of the first response (DA, −4334 ± 296 pA; and GA, −4177 ± 681 pA). *C*, ratio of *I_t_*_400_/*I_t_*_0_ for whole-cell recordings (WCR) and NMDAR EPSCs (*P < 0.05, ANOVA with Bonferroni multiple comparisons test).

### NMDAR current run-down is not reversed by a calmodulin-inhibitory peptide

It is well established in hippocampal neurones that Ca^2+^–calmodulin (Ca^2+^–CaM) binding to the GluN1 subunit C-terminal domain can induce Ca^2+^-dependent inactivation of NMDARs (Ehlers *et al*. 1996; Zhang *et al*. 1998; Rycroft & Gibb, 2002). To determine whether the Ca^2+^-dependent run-down in dopaminergic neurones was mediated by Ca^2+^–CaM, the protocol was repeated but with either a CaM-inhibitory peptide, based on the CaM-binding domain of MLCK (RRKWQKTGHAVRAIGRL, 50 μm; Calbiochem), or a control peptide with several amino acid substitutions (RRKEEKTGHAVRAIGRE, 50 μm; Calbiochem) included in the pipette solution. With 0.6 mm EGTA in the pipette solution (to optimize activation of Ca^2+^–CaM), there was no difference in the extent of whole-cell run-down between inhibitory (*I_t_*_400_/*I_t_*_0 _= 0.39 ± 0.02, *n* = 8) and control peptide (*I_t_*_400_/*I_t_*_0 _= 0.35 ± 0.03, *n* = 8; *P* = 0.37, Student's unpaired *t* test; Table S1). To ensure that slow dialysis was not preventing the peptide from reaching its target, the experiments were repeated but with a 20 min wait in whole-cell recording mode prior to the first NMDA application. Again, no significant differences were observed (Table S1).

Calcineurin is a Ca^2+^-dependent protein phosphatase that regulates NMDAR function (Tong *et al*. 1995; Krupp *et al*. 2002; Rycroft & Gibb, 2004b). Inclusion of the calcineurin inhibitor ciclosporin (500 nm) in the pipette solution with 0.6 mm EGTA had no significant effect on current run-down when compared with 0.6 mm EGTA alone (Table S1).

The NMDA current run-down in hippocampal neurones in culture involves actin depolymerization (Rosenmund & Westbrook, 1993a); therefore, run-down was tested in the presence of phalloidin (10 μm). Phalloidin was unable to reduce run-down (Table S1). These results do not support a role for CaM, calcineurin or actin depolymerization in mediating Ca^2+^-dependent run-down in substantia nigra dopaminergic neurones and suggest that different Ca^2+^-dependent NMDAR regulatory mechanisms operate compared with those reported in hippocampal neurones.

### Dynamin-dependent regulation

In hippocampal neurones, agonist binding can lead to activity-dependent changes in NMDAR trafficking, whereby receptors are internalized (Vissel *et al*. 2001; Li *et al*. 2002). A well-established form of NMDAR internalization is through clathrin-mediated endocytosis (Lavezzari *et al*. 2003), a process which is dependent on the GTPase dynamin but not on Ca^2+^ (Vissel *et al*. 2001; Nong *et al*. 2003; Montgomery *et al*. 2005). This has not been tested in dopaminergic neurones. As shown in Fig. 2, when elevations in intracellular Ca^2+^ were minimized by voltage clamping to +40 mV or replacing extracellular Ca^2+^ with Ba^2+^ and chelating intracellular Ca^2+^ with 10 mm BAPTA, a use-dependent decline in peak current was still observed (Fig. 2*E*). To test whether this run-down might be dynamin dependent, a dynamin-inhibitory peptide (QVPSRPNRAP; 50 μm; Tocris) was included in the intracellular solution, along with 10 mm BAPTA. Recordings of whole-cell NMDAR currents at +40 mV using a control peptide (QPPASNPRVR; 50 μm; Tocris; e.g. Fig. 5*A*) showed significant run-down (*I_t_*_400_/*I_t_*_0_, 0.80 ± 0.05; *n* = 8; *P* = 0.004, Student's paired *t* test), but no significant run-down was observed when using dynamin-inhibitory peptide (e.g. Fig. 5*B*; *I_t_*_400_/*I_t_*_0_, 1.04 ± 0.07, *n* = 10; *P* = 0.65, Student's paired *t* test; Fig. 5*C* and *D*). A significant difference in run-down was observed between control and dynamin peptides (Fig. 5*C* and Table S1; *P* < 0.05, ANOVA with Bonferroni multiple comparisons test).

**Figure 5:**
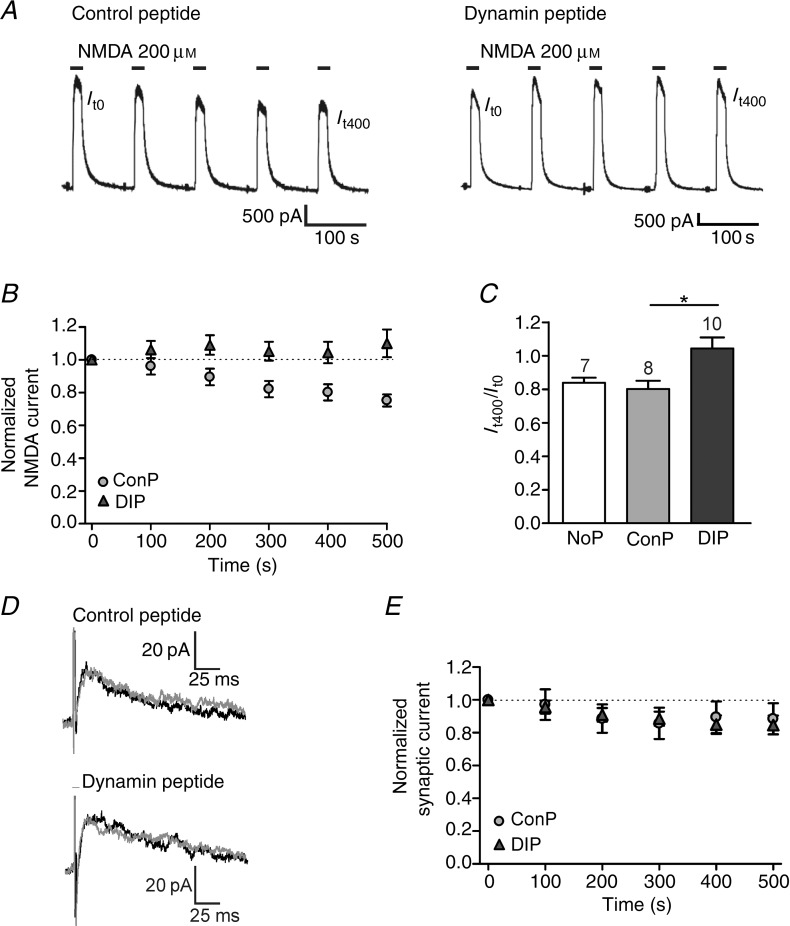
*A*, responses to repeated brief applications of 200 μm NMDA (glycine, 10 μm) applied every 100 s with 10 mm BAPTA and scrambled peptide (50 μm) or dynamin-inhibitory peptide (50 μm) in the pipette solution as indicated, from a neurone voltage clamped at +40 mV. *B*, time course of NMDAR current run-down with 10 mm BAPTA and either control peptide (ConP) or dynamin-inhibitory peptide (DIP) in the pipette solution. Current amplitudes were normalized to the amplitude of the first response. *C*, ratio of *I_t_*_400_/*I_t_*_0_ for NMDA applications every 100 s with no peptide (NoP), ConP or DIP in the pipette solution (*P < 0.05, ANOVA with Bonferroni multiple comparisons test). *D*, representative current trace of NMDAR EPSCs evoked by synaptic stimulation at 0.1 Hz in the presence of DNQX (10 μm), glycine (10 μm) and picrotoxin (50 μm). Responses were recorded at +40 mV with 10 mm BAPTA and control peptide (50 μm) or dynamin-inhibitory peptide (50 μm) in the pipette solution, as indicated. Traces are the average of 10 EPSCs. In black is the average of responses between 0 and 90 s. In grey is the average of responses between 400 and 490 s. *E*, changes in NMDAR-EPSC amplitude over time. Each time point represents the average of 10 EPSCs and is normalized to the average amplitude of the first response.

The NMDARs are internalized via clathrin-coated pits, which in hippocampal neurones are located peri-or extrasynaptically (Petralia *et al*. 2003; Newpher & Ehlers, 2009). It has been reported that synaptic NMDARs are more stable within the cell membrane than their extrasynaptic counterparts and less sensitive to use-dependent internalization (Li *et al*. 2002). In order to assess the stability of NMDARs at synaptic sites in dopaminergic neurones, NMDAR EPSCs were recorded at +40 mV. No significant run-down was detected with either peptide, and there was no significant difference between control and dynamin-inhibitory peptide (*P* = 0.72, Student's unpaired *t* test; Fig. 5*E* and Table S1). Although intracellular calcium dynamics are likely to be different between whole-cell and synaptic receptor activation protocols, these results suggest that during localized activation, synaptic NMDARs undergo less internalization by clathrin-mediated (dynamin-dependent) endocytosis than their extrasynaptic counterparts.

### Can agonist binding induce receptor modulation independent of ion flux?

Agonist binding can lead to modulation of the receptor response even in the absence of ion flux through the channel (Vissel *et al*. 2001). To determine whether this mechanism contributes to run-down in dopaminergic neurones, NMDA applications were made in conditions where ion flux through the NMDAR channel was blocked. As illustrated in Fig. 6*B*, an initial response was recorded in Mg^2+^-free solution at +40 mV followed by three agonist applications at −60 mV in the presence of 10 mm Mg^2+^. A test response was then recorded at +40 mV and the amplitude compared with the initial response. In these conditions, there was no significant run-down (*I_t_*_400_/*I_t_*_0_, 0.95 ± 0.07; *n* = 12; *P* = 0.1844). However, when no agonist was applied between the initial and test responses in the same conditions (e.g. Fig. 6*A*) the *I_t_*_400_/*I_t_*_0_ was significantly greater (1.20 ± 0.10; *n* = 9; *P* = 0.041, Student's unpaired *t* test; Fig. 6*C*), suggesting that agonist binding might trigger some run-down independently of ionic flux.

**Figure 6:**
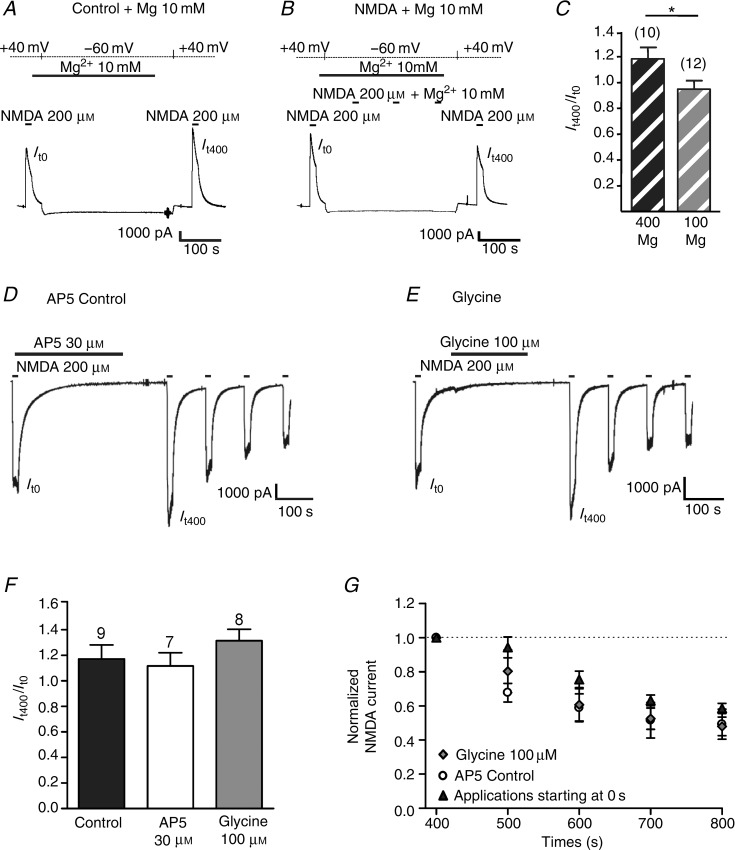
*A*, responses to repeated brief agonist applications of NMDA (200 μm; glycine, 10 μm) applied every 400 s, from a neurone voltage clamped to +40 mV. The slice was perfused with 10 mm Mg^2+^ at −60 mV between agonist applications. *B*, as in *A*, but with agonist applications made every 100 s during perfusion with 10 mm Mg^2+^ at −60 mV. *C*, ratio of *I_t_*_400_/*I_t_*_0_ for the control and test protocols described in *A* and *B* (*P = 0.04, Student's unpaired *t* test). *D*, responses to repeated applications of 200 μm NMDA (glycine 10 μm) from a neurone voltage clamped to −60 mV. The ‘conditioning stimulus’ was 30 μm AP5, applied for 285 s. *E*, as in *A*, but with 100 μm glycine as the conditioning stimulus. *F*, ratio of *I_t_*_400_/*I_t_*_0_ for the control (applications every 400 s, Fig. 1*C*), 30 μm AP5 and 100 μm glycine groups (not significantly different, ANOVA). *G*, time course of run-down following the NMDA application at 400 s for the AP5 (as in Fig. 6*D*) and glycine groups (as in Fig. 6*E*). For comparison, the time course of run-down is also shown for the experiment in which NMDA applications started at 0 s (Fig. 1*A*). All experiments were performed with 10 mm BAPTA in the pipette solution.

In rat hippocampal slices, a prolonged application of a high concentration of glycine to neurones can induce a robust decrease in NMDAR currents when receptors are subsequently activated with both NMDA and glycine (Nong *et al*. 2003). Glycine binding alone was found to be sufficient to produce an intracellular signal, ‘priming’ NMDARs for clathrin-mediated internalization (Nong *et al*. 2003). Here, a prolonged application of a high concentration of glycine (100 μm for 200 s) between receptor activation at *I_t_*_0_ and *I_t_*_400_ was used as a priming conditioning stimulus in an attempt to promote a more robust internalization of NMDARs in dopaminergic neurones (e.g. Fig. 6*E*). As a control, the glutamate binding site competitive NMDAR antagonist AP5 (30 μm) was applied instead of glycine (e.g. Fig. 6*D*). When glycine was used as the conditioning stimulus, the *I_t_*_400_/*I_t_*_0_ ratio (1.31 ± 0.10, *n* = 8) was not significantly different from that seen in the presence of AP5 (1.12 ± 0.10, *n* = 7; ANOVA; Fig. 6*F*). These results suggest that glycine priming was unable to cause an immediate decline in *I*_NMDA_ upon NMDA application, a finding that is in agreement with the results of Nong *et al*. (2003). In order to address whether glycine priming accelerates any subsequent decline in *I*_NMDA_, NMDA applications were made at 100 s intervals following measurement of *I_t_*_400_. The time course of run-down was very similar following either control (AP5) or glycine as the conditioning stimulus (Fig. 6*G*). The *I_t_*_800_/*I_t_*_400_ was 0.49 ± 0.09 with control (AP5) and 0.48 ± 0.05 with glycine (*P* = 0.1757, Student's unpaired *t* test). These results indicate that, unlike the situation in hippocampal neurones (Nong *et al*. 2003), in SNc dopaminergic neurones glycine priming was unable to either induce or enhance *I*_NMDA_ run-down.

## Discussion

NMDA receptor current run-down is a form of activity-dependent NMDAR regulation (Legendre *et al*. 1993; Rosenmund & Westbrook, 1993a,*b*1993b; Medina *et al*. 1996; Zhang *et al*. 1998). It can involve both changes in channel open probability and/or changes in the number of receptors on the cell surface and can control the contribution of NMDARs to synaptic signalling (Tong *et al*. 1995). Run-down might also be regarded as a protective mechanism to limit excessive Ca^2+^ influx during periods of high NMDAR activity (Hashimoto *et al*. 2002) at synaptic and extrasynaptic locations. The sensitivity to excitotoxic damage may be determined by a number of factors, such as the NMDAR subunit profile (Liu *et al*. 2007; Martel *et al*. 2012), the surface localization of the receptor (Hardingham & Bading, 2010), intracellular Ca^2+^ buffering (Rintoul *et al*. 2001), receptor association with scaffolding proteins (Forder & Tymianski, 2009) and, perhaps, activity-dependent receptor regulation. The aim of the present study was to investigate activity-dependent NMDAR current run-down in SNc dopaminergic neurones.

### Whole-cell NMDAR current run-down is in part Ca^2+^ dependent

Regular whole-cell NMDA applications to dopaminergic neurones induced large whole-cell currents that declined with each successive agonist application in a similar way to that described in other studies of NMDARs in hippocampal neurones (MacDonald *et al*. 1989; Rosenmund & Westbrook, 1993a,*b*1993b). This decline in peak current, or run-down, occurred only when repeated agonist applications were made (at 100 s intervals). This would suggest that run-down is due to receptor activity, rather than to dialysis of cytosolic components that might be a result of whole-cell recording or to release of Ca^2+^ from intracellular stores. Responses that ran down did not recover when agonist applications were withdrawn for 5 min, suggesting that in the conditions of whole-cell patch-clamp recording, the decrease in NMDAR activity was long lasting rather than transient.

The extent of whole-cell NMDAR current run-down was increased by reducing intracellular Ca^2+^ buffering; NMDAR run-down was greater when neurones were dialysed with 0.6 mm EGTA (estimated free Ca^2+^, ∼735 nm) than with 10 mm BAPTA (estimated free Ca^2+^, ∼20 nm). EGTA and BAPTA have a similar binding affinity for Ca^2+^, but markedly different binding kinetics, with BAPTA being able to sequester Ca^2+^ in the region of two orders of magnitude faster than EGTA (Pethig *et al*. 1989; Adler *et al*. 1991). Buffers dialysed into a cell are able to compete for Ca^2+^ binding with endogenous Ca^2+^-binding proteins. The difference in run-down observed with the two buffers in the experiments presented here suggests that the Ca^2+^-dependent proteins that regulate NMDAR run-down in dopaminergic neurones are able to bind Ca^2+^ more rapidly than EGTA but less rapidly than BAPTA.

We have previously reported that NMDARs in SNc dopaminergic neurones are composed of GluN2B and GluN2D subunits, most probably in a combination of di-and tri-heteromers (Jones & Gibb, 2005; Brothwell *et al*. 2008; Suárez *et al*. 2010). To examine the subunit dependence of run-down, the run-down was measured after incubation with the GluN2B-preferring antagonist, ifenprodil. Significant run-down of the ifenprodil-insensitive NMDAR component was observed, but showed no sensitivity to intraceullar Ca^2+^ chelator. This is consistent with the ifenprodil-insensitive component being GluN2D mediated, because GluN2D currents show little desensitization (Wyllie *et al*. 1998).

The source of Ca^2+^ appears to be primarily extracellular, because substitution of extracellular Ca^2+^ with Ba^2+^ and reducing the Ca^2+^ driving force by clamping neurones at +40 mV both caused a significant reduction in run-down (Fig. 2). However, a role for Ca^2+^ release from intracellular stores cannot be excluded. While Ba^2+^ is able to permeate the NMDAR channel pore (Mayer & Westbrook, 1987; Ascher & Nowak, 1988), it does not activate Ca^2+^-dependent proteins (Chao *et al*. 1984; Richardt *et al*. 1986). Extracellular substitution of Ca^2+^ with Ba^2+^ is known to reduce (but not prevent) NMDAR inactivation (defined as the decline in peak current observed during a single application of agonist) in hippocampal neurones and in transfected HEK 293 cells (Legendre *et al*. 1993; Krupp *et al*. 1996) and slow the decay kinetics of EPSCs in cultured cortical neurones (Umemiya *et al*. 2001), probably due to a reduction in the kinetic effects of Ca^2+^–CaM binding to the receptor (Rycroft & Gibb, 2002).

Given the prominent role that CaM is known to play in regulating NMDARs (Rosenmund & Westbrook, 1993a; Ehlers *et al*. 1996; Krupp *et al*. 1999; Rycroft & Gibb, 2002; reviewed by Lisman *et al*. 2012), it is perhaps surprising that the CaM-inhibitory peptide had no effect on Ca^2+^-dependent current run-down in our experiments. Furthermore, our data suggest that neither calcineurin nor alpha-actinin-cytoskeletal interactions mediate Ca^2+^ dependent rundown. In SNc dopaminergic neurones, other Ca^2+^ dependent molecules, such as gelsonlin, may be important (Furukawa *et al*., 1997).

### Whole-cell NMDAR current run-down is greater in SNr than in SNc

Nigrostriatal dopaminergic neurones are known to degenerate in Parkinson's disease, while GABAergic neurones of the SNr are much less affected (Braak *et al*. 2003). The changes in the circuitry of the basal ganglia that occur following nigrostriatal degeneration include an increase in activity of the subthalamic nucleus, which is a major source of glutamatergic input to both the SNc and the SNr (Hamani *et al*. 2004; Watabe-Uchida *et al*. 2012). This enhanced excitatory input is hypothesized to contribute to excitotoxic neuronal damage to dopaminergic neurones in Parkinson's disease (Piallat *et al*. 1996, 1999; Bezard *et al*. 1999). If this alone were the pathological trigger, then GABAergic neurones might also be vulnerable, but pathological changes such as mitochondrial dysfunction and an increase in reactive oxygen species might enhance excitotoxicity in a way that is unique to dopaminergic neurones in Parkinson's disease (reviewed by Schapira, 2008; Surmeier *et al*. 2010). Consistent with our previous work (Suárez *et al*. 2010), in these experiments NMDAR current run-down was greater in SNr GABAergic neurones than in SNc dopaminergic neurones with BAPTA in the pipette solution. It is possible that differences in regulatory mechanisms exist between the two neuronal populations, such as differences in Ca^2+^-binding proteins or receptor trafficking pathways. As run-down may be a protective mechanism to limit excessive Ca^2+^ influx during periods of high activity (Hashimoto *et al*. 2002), the more pronounced run-down in SNr GABAergic neurones might reflect a more robust protective response. This may in turn affect the sensitivity of the two neuronal populations to excitotoxic stress caused by enhanced glutamatergic input from the subthalamic nucleus in Parkinson's disease (Hamani *et al*. 2004).

In the present study, we investigated the regulatory mechanisms present in dopaminergic neurones using rats aged ∼7 days postnatal as a model system. Our previous pharmacology experiments suggested only small changes in NMDA receptor subunit composition between postnatal days 7 and 28, because the proportions of both synaptic current and whole-cell NMDA current blocked by ifenprodil are quite similar between postnatal days 7 and 28 (Brothwell *et al*. 2008; Suárez *et al*. 2010). This suggestion agrees well with the results of earlier biochemical experiments showing that receptor subunit expression in the developing and adult rat midbrain is dominated by GluN2B and GluN2D (Monyer *et al*. 1994; Dunah *et al*. 1996; Wenzel *et al*. 1996). A more subtle question is whether, in mature rats, the proportion of triheteromeric GluN1/GluN2B/GluN2D receptors (Dunah *et al*. 1998) is significantly different from that early in development. More selective pharmacological tools are needed to address this question. However, it would be interesting to investigate whether similar mechanisms of NMDA receptor run-down are apparent in ageing rats and in rodent models of Parkinson's disease.

### Calcium-independent regulation of whole-cell NMDAR current run-down

The persistence of a small degree of whole-cell current run-down in conditions where Ca^2+^ influx was minimized suggested that, like NMDARs in hippocampal neurones (Vissel *et al*. 2001; Li *et al*. 2002), those in SNc dopaminergic neurones might also be regulated by Ca^2+^-independent mechanisms. A change in peak current was observed when ion flux through the receptor was blocked with Mg^2+^, suggesting that a reduction in the surface expression of NMDARs, independent of ion flux, may occur in SNc dopaminergic neurones; these receptors are composed of GluN2B and GluN2D subunits (Dunah *et al*. 1998; Jones & Gibb, 2005; Brothwell *et al*. 2008; Suárez *et al*. 2010). Endocytosis of GluN2B-containing receptors has been studied extensively and is regulated by phosphorylation of a tyrosine residue found in the YEKL motif at the distal end of the C-terminal region (Roche *et al*. 2001; Lavezzari *et al*. 2004; Scott *et al*. 2004; Prybylowski *et al*. 2005; Zhang *et al*. 2008). It seems plausible that GluN2B-containing receptors in SNc dopaminergic neurones are partly regulated in a Ca^2+^-independent, activity-dependent manner that might reflect changes in tyrosine phosphorylation and receptor surface expression. However, glycine ‘priming’ of this mechanism, as seen in hippocampal neurones (Nong *et al*. 2003), was not observed in dopamine neurons (Fig. 6*G*).

Activity-dependent changes in NMDAR trafficking in SNc dopaminergic neurones were further substantiated by the reduction in current run-down during intracellular dialysis with a dynamin-inhibitory peptide, suggesting that run-down is in part due to removal of NMDARs from the neuronal membrane. This is consistent with previous findings that GluN2B-containing receptors are internalized by clathrin-mediated endocytosis (Roche *et al*. 2001; Lavezzari *et al*. 2004; Scott *et al*. 2004; Prybylowski *et al*. 2005; Zhang *et al*. 2008) and that internalization can be regulated in an activity-dependent manner (Vissel *et al*. 2001; Li *et al*. 2002).

### Regulation of synaptic NMDAR current run-down

The strength of synaptic NMDAR signalling can also be regulated in a Ca^2+^-dependent manner in cultured hippocampal (Rosenmund *et al*. 1995; Tong *et al*. 1995; Mennerick & Zorumski, 1996; Medina *et al*. 1999) and cortical neurones (Umemiya *et al*. 2001). Repeated low-frequency stimulation of autaptic cultured hippocampal neurones decreased the amplitude of NMDA EPSCs in a Ca^2+^-dependent manner (Rosenmund *et al*. 1995), and the decay kinetics of miniature NMDA EPSCs was altered by intracellular perfusion with BAPTA but not EGTA (Umemiya *et al*. 2001).

In the data presented here, NMDA synaptic current run-down was observed in response to repeated low-frequency synaptic stimulation (0.1 Hz) in neurones voltage clamped to −50 mV (Fig. 3). This was not altered by changing the strength of intracellular Ca^2+^ buffering (i.e. replacing 0.6 mm EGTA with 10 mm BAPTA), but run-down was significantly reduced by clamping neurones to +40 mV, which reduces the driving force for Ca^2+^ influx. The lack of sensitivity of synaptic NMDAR current run-down to the Ca^2+^ chelators, BAPTA *versus* EGTA, in contrast to whole-cell NMDAR current run-down, may be due to the mode of NMDAR activation (rapid and brief *versus* slower and more prolonged) or due to a much closer coupling of the Ca^2+^-dependent mechanism of run-down to synaptic NMDARs, such that even the fast chelator BAPTA is not able to sequester the rapid Ca^2+^ influx effectively through the synaptic NMDARs.

In cultured hippocampal neurons, trafficking of synaptic and extrasynaptic NMDARs is differentially regulated, with synaptic receptors being less sensitive to internalization than their extrasynaptic counterparts (Li *et al*. 2002). The NMDA EPSCs in dopamine neurons were not affected by intracellular dialysis with a dynamin-inhibitory peptide, suggesting there is little receptor internalization in these conditions. The stability of synaptic NMDARs in other neuronal populations is attributed to association with scaffolding molecules, such as PSD-95, in the postsynaptic density (Rao *et al*. 1998; Roche *et al*. 2001; Lim *et al*. 2003; Prybylowski *et al*. 2005; Groc *et al*. 2009) and association with tyrosine kinases (Goebel-Goody *et al*. 2009), both of which inhibit NMDAR endocytosis (Lavezzari *et al*. 2003). It may be that synaptic NMDARs in SNc dopaminergic neurones also form a stable receptor pool.

In summary, NMDARs in SNc dopaminergic neurones are robustly regulated in an activity-dependent manner. Evidence has been presented here for both Ca^2+^-dependent run-down of synaptic and whole-cell NMDAR currents and Ca^2+^-independent changes in whole-cell receptor trafficking. These mechanisms may contribute to intracellular Ca^2+^ homeostasis in dopaminergic neurones by limiting Ca^2+^ influx through the NMDAR and are therefore a possible mechanism of dysregulation in Parkinson's disease.

## Key points

- Calcium ion influx through *N*-methyl-d-aspartate receptors (NMDARs) may contribute to substantia nigra pars compacta (SNc) dopaminergic neurone dysfunction in Parkinson's disease.
- Responses of NMDARs in dopaminergic neurones showed use-dependent run-down that was not readily reversible and was partly dependent on Ca^2+^ influx and partly dependent on clathrin-mediated endocytosis.
- Thus, we report regulation of NMDARs in SNc dopaminergic neurones by intracellular Ca^2+^ at both synaptic and extrasynaptic sites and provide evidence for activity-dependent changes in receptor trafficking.

## Abbreviations

AP5: D-2-amino-5-phosphonovaleric acid
BAPTA: 1,2-bis(o-aminophenoxy)ethane-N,N,N′,N′-tetraacetic acid
CaM: calmodulin
DA: Dopaminergic
DNQX: 6,7-dinitro-quinoxaline-2,3-dione
EGTA: Ethylene glycol-bis(2-aminoethylether)-N,N,N′,N′-tetraacetic acid
EPSC: excitatory post synaptic current
GA: GABAergic
GluN2A: NMDA receptor subunit type 2A
GluN2B: NMDA receptor subunit type 2B
GluN2C: NMDA receptor subunit type 2C
GluN2D: NMDA receptor subunit type 2D
*I*_h_: hyperpolarization-activated inward current
I_NMDA_: NMDA activated current
MLCK: myosin light chain kinase
NMDAR: *N*-methyl-d-aspartate receptor
SNc: substantia nigra pars compacta
SNr: substantia nigra pars reticulata.
WCR: Whole cell recording
