# Supplementary material for: Activity-dependent regulation of NMDA receptors in substantia nigra dopaminergic neurones
Source: J Physiol. 2014 Jan 31;592(Pt 4):653–68. doi: 10.1113/jphysiol.2013.267310 (PMC3934707; doi:10.1113/jphysiol.2013.267310)
Supplement: Supplementary file 1 [file tjp0592-0653-sd1.doc]

|  | **n** | **Initial amplitude t_0s_ (pA)** | | | **Amplitude t_400s_ (pA)** | | | **Ratio**  **It_400s_/ It_0s_** |
| --- | --- | --- | --- | --- | --- | --- | --- | --- |
| **Whole-cell NMDA** |  | | | | | | | |
| EGTA, -60 mV | 15 | 4644 ± 462 | | 1653 ± 219 | | | 0.37 ± 0.02 | |
| CaM inhibitory peptide | 8 | 4792 ± 704 | | 1872 ± 304 | | | 0.39 ± 0.02 | |
| CaM inhibitory peptide (+20min)^1^ | 4 | 4663 ± 405 | | 1831 ± 104 | | | 0.4 ± 0.04 | |
| CaM control peptide | 8 | 5063 ± 558 | | 1693 ± 143 | | | 0.35 ± 0.03 | |
| Cyclosporin A | 6 | 5482 ± 497 | | 1917 ± 246 | | | 0.35 ± 0.35 | |
| BAPTA, -60 mV | 16 | 4334 ± 296 | | 2258 ± 235 | | | 0.59 ± 0.03 | |
| Phalloidin *** | 5 | 5412 ± 460 | | 1632 ±122 | | | 0.33 ± 0.04 | |
| BAPTA, +40 mV | 7 | 3991 ± 431 | | 3297 ± 386 | | | 0.85 ± 0.06 | |
| Dynamin inhibitory peptide * | 10 | 3751 ± 236 | | 3870 ± 264 | | | 1.04 ± 0.07 | |
| Dynamin control peptide | 8 | 3662 ± 401 | | 3029 ± 501 | | | 0.80 ± 0.04 | |
| **NMDA-EPSCs** |  | | | | | | | |
| EGTA, -50 mV | 8 | | 54 ± 7 | 34 ± 3 | | 0.67 ± 0.08 | | |
| CaM inhibitory peptide | 8 | | 43 ± 2 | 34 ± 3 | | 0.79 ± 0.04 | | |
| CaM control peptide | 8 | | 49 ± 7 | 37 ± 5 | | 0.70 ± 0.03 | | |
| BAPTA, -50 mV | 10 | | 67 ± 8 | 37 ± 4 | | 0.58 ± 0.05 | | |
| Phalloidin | 8 | | 57 ± 13 | 32 ± 5 | | 0.65 ± 0.11 | | |
| BAPTA, +40 mV | 10 | | 50 ± 8 | 45 ± 9 | | 0.86 ± 0.06 | | |
| Dynamin inhibitory peptide | 6 | | 48 ± 11 | 38 ± 6 | | 0.85 ± 0.06 | | |
| Dynamin control peptide | 8 | | 65 ± 9 | 55 ± 6 | | 0.89 ± 0.10 | | |

**Supplemental Table 1.** Modulation of NMDAR rundown by intracellular factors.

^1^CaM was allowed to diffuse into the cell for 20 min before recording the NMDA responses

**P* < 0.05 compared with dynamin control peptide (ANOVA)

****P* = 0.0002 compared with BAPTA at -60 mV alone, unpaired *t*-test
